# How Baccalaureate Nursing Students Value an Interprofessional Patient Safety Course for Professional Development

**DOI:** 10.5402/2012/401358

**Published:** 2012-03-14

**Authors:** Amy A. Abbott, Kevin T. Fuji, Kimberly A. Galt, Karen A. Paschal

**Affiliations:** ^1^School of Nursing, Center for Health Services Research and Patient Safety, 2500 California Plaza, Omaha, NE 68178, USA; ^2^Center for Health Services Research and Patient Safety, 2500 California Plaza, Omaha, NE 68178, USA; ^3^School of Pharmacy and Health Professions, Center for Health Services Research and Patient Safety, 2500 California Plaza, Omaha, NE 68178, USA

## Abstract

Nursing students need foundation knowledge and skills to keep patients safe in continuously changing health care environments. A gap exists in our knowledge of the value students place on interprofessional patient safety education. The purpose of this exploratory, mixed methods study was to understand nursing students' attitudes about the value of an interprofessional patient safety course to their professional development and its role in health professions curricula. Qualitative and quantitative data were collected from formative course performance measures, course evaluations, and interviews with six nursing students. The qualitative themes of awareness, ownership, and action emerged and triangulated with the descriptive quantitative results from student performance and course evaluations. Students placed high value on the course and essential nature of interprofessional patient safety content. These findings provide a first step toward integration of interprofessional patient safety education into nursing curricula and in meeting the Institute of Medicine's goals for the nursing profession.

## 1. Introduction

Despite a decade of focused national attention, health care delivery is not safer for patients across the nation [1]. Leaders in health care and policy collectively have called for preparing health care professionals who are able to effect patient care systems improvement through interprofessional collaboration in order to improve patient safety. The Institute of Medicine (IOM) [2] report “The Future of Nursing” emphasizes that “all health professionals should receive more of their education in concert with students from other disciplines.” Interprofessional team training of nurses, physicians, and other health care providers should begin when they are students and proceed throughout their careers. Interprofessional education is based on the premise that students who are more familiar with and respect one another's roles and contributions to the interprofessional team are critical to realizing the full potential of nursing [3]. Additionally, interprofessional education fosters collaboration in the workplace and allows problem solving that exceeds the capacity of any one profession. Although the literature is growing in the nursing profession about courses/curriculum and professional formation in patient safety, nursing students in the United States continue to be educated in the context of discipline-specific activities rather than via an interdisciplinary systems approach [4, 5]. Educators continue to struggle on how to include interprofessional education in an already jam-packed curriculum without it appearing as an “add-on” versus an integral and necessary component. Educating nursing students about patient safety through an interprofessional curriculum has the potential to prepare our future nursing professionals with the capacity envisioned by the Institute of Medicine.

“The Future of Nursing” report has identified key goals that the nursing profession must work toward in order to allow all members of the profession to achieve the full potential of their practice abilities. One of these goals addresses improved education systems that would promote the skills necessary for new nurses to collaborate and negotiate with an interprofessional team. While this approach is predominately absent in baccalaureate curricula in the United States, it is present in other countries such as Australia [6]. The advancing science of safety demands innovation in educational delivery by the academy to meet this educational responsibility. The IOM report “Health Professions Education: A Bridge to Quality” provides guidance for the core competencies needed in health professions curricula to improve quality and safety in health care [5]. These competencies were developed to provide a consistent framework for interprofessional education [7, 8]. Professional associations and specialized accrediting bodies in the health professions began revising their standards in 2000 to explicitly recognize curricular movements in patient safety [9]. The nursing profession now has clear educational expectations through the *Nursing Scope and Standards of Practice* [10] and the *Essentials of Baccalaureate Education for Professional Nursing Practice *[11] to address the IOM goals. The American Association of Colleges of Nursing is leading the national effort to meet these goals through the Quality and Safety Education for Nurses (QSEN) project. This project was funded through the Robert Wood Johnson Foundation to enhance the ability of nursing faculty to effectively develop quality and safety competencies to prepare graduates with the knowledge, skills, and attitudes necessary to continuously improve the quality and safety of the health care systems within which they work [12]. The project focuses on the six key areas of patient-centered care, teamwork and collaboration, evidence-based practice, quality improvement, safety, and informatics.

Similarly, in 2005, Creighton University developed an interprofessional course, teaching core concepts in the foundations of patient safety in the context of interprofessional collaboration to deliver patient-centered care [13]. The course was intentionally designed for explicit interprofessional education of baccalaureate nursing students with other students in the health professions. Patient safety provides an optimal opportunity for interprofessional learning and interaction to begin at the student-level and translates to better interpersonal communications in the clinical setting. Foundational knowledge about the science of patient safety is applicable across all patient care settings and is increasingly necessary for navigating patients a cross-multiple settings and teams of health care professionals.

However, little is known about how health professions students perceive this content [14]. How do nursing students embrace patient safety in their professional identity? What are their attitudes toward their own professional formation in the context of interprofessional education? Professional development in the context of interprofessional patient-centered care is key to ensuring that care is continuous, reliable, and safety focused [4]. Nurses are vital in the delivery of safe care and often integral to interdisciplinary communication during patient care. Educators must prepare students with foundational knowledge about how to provide patient centered, interdisciplinary, safe care in continuously changing health care environments. There is a need to understand the effectiveness of this model of educational delivery into the nursing student's professional learning and how nursing students perceive the contribution of interprofessional education in their own professional development.

## 2. Materials and Methods

### 2.1. Course Description

The interprofessional patient safety course, *Foundations in Patient Safety*, developed at Creighton University, was recognized by the IOM as one of the nation's first interprofessional patient safety courses [15]. This elective course is offered yearly and is open to all students across the university. The course is team taught by interprofessional faculty from nursing, medicine, pharmacy, law, physical therapy, social work, decision sciences, bioethics, and health care administration. This interactive, didactic course is block taught over four weeks and focuses on content mastery of patient safety science rather than discipline-specific measures. Additional information on course development, design, and instruction are detailed in a previous publication [13] and the content of the course can be found in the textbook coedited and written by the course faculty, including the study authors [16].

### 2.2. Contextual Theory

The early curricular introduction of the interprofessional patient safety course to health professions students is founded on the premise that early professional identity formation for students must include knowledge and valuing the role of patient safety sciences in professional practice. The interprofessional exposure occurs with other health professionals during professional formation. In this sense, professional formation is understood as a relationship between student learning experiences and the way in which they anticipate practicing in their professional work lives [17]. The nursing profession has explicitly recognized that knowledge in all three domains (cognitive, psychomotor and affective) is essential for full professional development and socialization into the profession. Affective domain learning is linked to value formation and the embodiment of professional value [18].

The instructional design approach to the course involves classroom-based pedagogies of engagement. Both cooperative-group learning and problem-based learning governed the design of in-classroom learning experiences. The faculty considered to what extent an idea, topic, or process in the context of patient-centered interdisciplinary patient safety learning: (1) represented a big idea and had sustained value beyond the classroom; (2) was at the heart of the interdisciplinary patient safety sciences; (3) required further development in students; (4) had the potential for engaging students [19].

### 2.3. Research Questions and Study Purpose

The purpose of this study was to explore nursing students' attitudes about the value of an interprofessional patient safety education course to their own professional formation. The study was guided by the central research question: how do nursing students value patient safety in their own professional development when learned through an interprofessional context? This question was studied in the context of examining the nursing students' academic performance in the course, both individually and comparatively with all students' course performance from the various disciplines of study enrolled at the time. To guide this understanding, two research subquestions were posed. (1) Is there a difference between nursing student performance as compared to the overall group of students in the course? (2) How are nursing students' attitudes towards their own professional development understood in the context of all students' course performance?

### 2.4. Design and Methods

**Figure 1:**
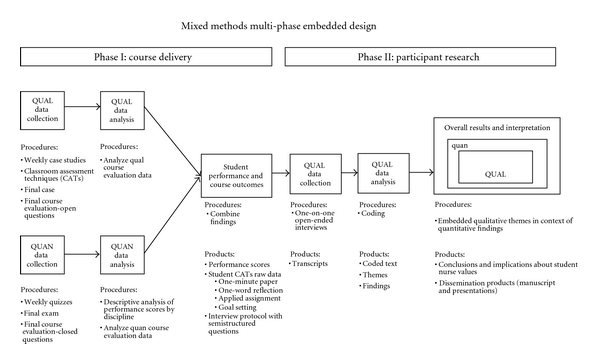
Mixed methods multi-phase embedded design.

 An exploratory, mixed methods, and embedded design study was conducted during the Spring 2009 course offering [20]. Using this type of design, different but complementary data on the same topic are collected to best understand the central phenomenon of the study, nursing students' attitudes toward an interprofessional patient safety course. This design draws on the strengths of qualitative and quantitative methodologies to produce logical generalization of the results [21] (see Figure 1).

Critical case sampling was used to recruit nursing students following course completion and final grade notification. Students who expressed willingness to an interview as indicated by their response to a question on the course evaluation were contacted directly. Interviews were conducted with six consenting nursing students of the nine who enrolled this semester. All of the nursing students were in their second year of nursing school and did not have formal clinical rotations. Half were employed in the clinical setting either as a nursing assistant or as a unit secretary. This study was approved by the university's institutional review board which reviews and assesses all research projects for acceptable standards relating to protection of human subjects.

 Students participated in a 4-week interprofessional course. Course content focused on (1) providing students with foundational knowledge about the national patient safety problem; (2) using a systems approach to improving patient safety; (3) developing a culture of safety; (4) identifying and using different tools to improve safety in their practice setting (e.g., root cause analysis); (5) understanding why errors and mistakes happen and how to handle them.

Each class session began by introducing patient safety concepts through the use of videos and/or cases. This design was used to accelerate student engagement in course content by emoting a response from students by bringing the clinical environment into the classroom. This quickly drew the students into the content of allowing them to make meaning of the concepts presented in the context of their professional learning and personal experience with error. Students were invited to share their comments and feelings about what they watched and/or heard. This process sparked further discussion with multiple professionals adding their respective insightful contributions allowing those in another profession to gain perspective on different roles and responsibilities.

 Students were encouraged to individually embrace discipline-specific content and transfer this learning from the interprofessional experience to their own personal and professional practice roles. This learning occurred within the context of interprofessional practice as they developed knowledge of the science of patient safety. Part of this awareness developed as students began to understand the role of the other health professionals. Students engaged in interprofessional teamwork and communication to apply knowledge and skills learned to identify problems and generate solutions in complex case studies. Further development of these skills was promoted through individual learning assignments which required each student to make meaning of the classroom applications in a homework assignment.

### 2.5. Quantitative Data Collection and Analysis

 During the course, participants' perceptions regarding the importance of taking an interprofessional patient safety course were quantitatively explored, along with an assessment of student learning and understanding about the science of patient safety. Quantitative data were collected and analyzed using information from: (1) the 13-item standard course evaluation questionnaire given to all students enrolled in the course; (2) student performance on the final examination; (3) student performance on the final case study assignment; (4) the final course grade. The course evaluation was constructed with a primary stem and a 5-point Likert scale extent of agreement response (1 = strongly disagree to 5 = strongly agree). Five items from the 13-item course evaluation were selected for analysis. These items assessed students' attitudes about the basic principles and tenets of patient safety, patient safety as a professional practice framework, a systems approach to the science of patient safety, the development of interprofessional communication skills, and tools health professionals need to improve patient safety. The final examination consisted of 50 multiple-choice questions reflective of cumulative content presented throughout the course. The final case study incorporated numerous concepts, disciplines, and settings in the context of a patient safety scenario and required students to apply the learned content. The final course grade consisted of the weighted scores from weekly quizzes (40%), weekly and final case studies (40%), and the final examination (20%). Descriptive analysis was conducted using the Statistical Package for the Social Sciences software [22]. Student performance scores were extracted and overall performance evaluated. Final examination and final case study performance were compared to the individual's course evaluation to validate the quantitative findings. If students responded positively to the perception-based questions on the final course evaluation, they would also be expected to demonstrate a high level of content knowledge reflected by their performance on the final examination.

### 2.6. Qualitative Data Collection and Analysis

 The initial qualitative phase of the study was conducted concurrently with course instruction through a variety of classroom assessment techniques (CATs) and the course evaluation. This initial phase was designed to further our understanding of how nursing students thought the content presented in the course changed their approach to practicing their profession. The specific qualitative data collection methods were to have students: (1) generate a one-word response to viewing a video of a medical error; (2) identify and prioritize three learning outcome goals they wanted to accomplish during the course; (3) identify and describe a situation appropriate for root cause analysis (RCA); (4) prepare a one-minute paper on the student's beliefs about the importance of disclosure; (5) respond to open-ended questions on the course evaluation which assessed the students' perceptions of where the course content best fit in their professional curriculum and the value they placed on engaging with an interprofessional group of faculty and students.

Following course completion, the second phase of qualitative data was collected through interviews of nursing students. Interviews were conducted using a semistructured protocol focused on the central research question: what value do nursing students place on patient safety in their own professional development after engaging in an interprofessional patient safety course? The interviews were guided by individual responses to items on the course evaluation and by the three key ideas: (1) the role of the nursing profession, the role of the individual nurse and the role of the nursing student in the context of patient safety; (2) how skills and attitudes learned in the course will change their behavior as students and future practitioners; (3) recommendations for incorporation of patient safety content into the nursing curriculum. Each interview was audio recorded using an MP3 voice recorder, transcribed, reviewed for accuracy and completeness by the interviewer, and* in vivo* coded by each researcher. Overarching themes emerged from the data based on reading and coding by the individual researchers. This was followed with subsequent discussions, to achieve consensus on themes that best described the data. Following the analysis, key exemplars were selected to illustrate themes for use in dissemination of the findings.

### 2.7. Mixed Analysis

 Quantitative results formed the context for interpretation of qualitative results and provided a framework to position nursing students within the entire class based on performance. This is needed in an embedded mixed methods study as it allows for the primary dataset (qualitative findings) to be supported by the secondary dataset (quantitative) and results in a more complete picture. These results were triangulated with the qualitative themes that emerged from the interviews using a convergence model to draw valid conclusions about a phenomenon that is of nursing student attitudes towards an interprofessional patient safety course [20].

## 3. Results

### 3.1. Quantitative Findings

 The average overall grade in the course was 95.2%. There was no observable difference between nursing student performance and the rest of the class that would require unique interpretation of the findings. The mix of students in the class was as follows: 19 pharmacy students, 8 nursing students, and 1 law student. Students averaged 88.7% on the final case study, which was designed to assess students' abilities to comprehensively apply learned knowledge to a specific patient safety situation. On the final course examination, designed to assess students' knowledge and recall of core patient safety science content, students averaged 89.2%.

Students expressed positive views regarding content, knowledge gained, ability to apply knowledge, and their interactions with classmates and instructors. Students estimated that on average, 80% of course material was new knowledge that they had not learned elsewhere in their professional program curriculum. All students indicated that the content taught was either essential core knowledge or enriching to all health professionals.

### 3.2. Qualitative Findings

 Three themes emerged from the qualitative data analysis of the six nursing student interviews: *awareness, ownership,* and *action*.

*Awareness* of patient safety developed as students learned about the magnitude and severity of the problem, the foundational knowledge of patient safety science, and the meaning of patient safety to their role as a health care professional. During the first class session, students viewed the video, *beyond blame*, which describes several major medical errors involving multiple members of an interprofessional health care team and the impact these errors had on each professional. After watching the video, students were asked to respond with one-word reactions. These reactions evoked such descriptors as, “*shameful*,” “*scary,*” and “*shocking.”* Growing *Awareness *was also evident as students set individual learning outcome goals. One student wrote, “*Learn how patient safety became and is still a big issue, and why it is overlooked by many.*” On the final evaluation, students were asked to provide additional comments to help the course instructors understand the impact and value of this course. *Awareness *was reflected in comments such as, “*Just making people aware makes them more conscious of the impact they actually have on people's lives,” *and, *“Now that I have this knowledge and background in patient safety I will be more aware of things when I begin working.”*

 Students' reflections shared during interviews also demonstrated Awareness. Students expressed that, *“Patient safety is a lot you really do not think about”; “Mistakes aren't as rare as you think they are”; and, “Everyone's going to make a mistake.”* One student captured the essence of this theme when she said*, “It really is an eye opener and makes you aware of what really goes on out in the real world*.”

 The theme, *ownership*, is representative of students identifying the roles of both the individual and others in patient safety. During this process, there was identification of the role of the nurse as well as understanding and valuing the role of other health care providers. As students developed knowledge and skills in patient safety, they realized that patient safety is a key component of nursing care not only in the context of their individual profession, but also through their role as a member of an interprofessional team. The CAT responses from the one-minute paper on disclosure and its potential impact on their current and future practice indicated that students were taking ownership of their responsibility to provide safe patient care. This is reflected in a student's response as she defined her role as part of the solution.

*“This knowledge has prepared me in the future in terms of how to act when an error has occurred. I learned that disclosure is very important and can help improve interprofessional relationships. When it comes to ethics and morals, it is the right thing to do for the patient and the patient's family.*

*Reporting errors also helps health care professionals learn from these mistakes and work towards preventing these errors from happening again.”*

During interviews, one student summarized the concept of *ownership* by noting,* “…every step of the way there's going to be safety incorporated…it is part of me doing my job to make sure that the patient is safe.”*

 The final theme, *action*, emerged as students described discussing patient safety with others, advocating for patients, and ensuring safe patient care delivery. During the course, students used basic tools to act upon their *awareness* and *ownership*. During classroom time, there were small group exercises in which students were asked to collaborate and apply basic patient safety concepts they had learned. In response to a question on a CAT asking students to identify how this knowledge will impact how they will do their job when they are a practicing professional, one student responded, *“It gives me hope that I will be able to freely express and be willing to admit and take responsibility for the action that I have done.” Action *was also evident in the application of the course content in the final case study and during interviews. During her interview, one student said, *“When I was taking the class, I'd go to work and I'd be like, “Hey, I'm taking this class. This is what I learned today.” ”* This comment explicates both the desire to impart knowledge to others and empowerment to *action *signifying growth in both knowledge and attitudes related to the acquisition of that knowledge.

#### 3.2.1. Mixed Findings

 High scores on course performance measures reflected that students not only possessed content knowledge about the core science of patient safety but that they could apply this knowledge to interprofessional situations. On the final course evaluation, all students strongly agreed/agreed that they: (1) can apply basic principles of patient safety; (2) value patient safety as a professional practice framework; (3) can identify tools health professionals need to work effectively with the team to improve patient safety. All but two respondents strongly agreed/agreed that they developed interprofessional communication skills with common patient safety language. Data was triangulated using student comments from interviews, student performance evaluations, and responses to CATs with the identified qualitative themes of *awareness, ownership*, and *action *thereby increasing the reliability of the findings. Mixed analysis results suggest that students developed basic patient safety knowledge and the skills to incorporate this knowledge in future practice. As they developed awareness and gained knowledge, they took ownership for advancing patient safety within their professional framework and were empowered to take action in their present day situations. In order to validate the findings, interviewees were sent the results and asked to comment on whether or not the findings represented their views about the course. All responded that they could see themselves within these findings.

## 4. Discussion

Findings from this study support the “Future of Nursing” recommendations for interprofessional education. Nursing student participants demonstrated the value of interprofessional patient safety education in the context of their professional development. The progression of students as they gain an awareness of patient safety concepts and principles, to feeling ownership of their own professional responsibilities and acting upon those responsibilities within the context of interprofessional learning and interactions emphasize the potential for report recommendations to impact nursing education. In order to achieve the goals of the IOM, all nurses must possess foundational patient safety knowledge in order to ensure safe and high-quality patient care and they must have the teamwork skills to collaborate and negotiate with the interprofessional health care team [4, 23]. Nursing education in the United States will not be able to meet the goals of “The Future of Nursing” without providing foundational competency and experience in engaging with an interprofessional care team [24]. Nurses are at the core of changing the health care environment; therefore, nurse educators must provide students with the knowledge, skills, and attitudes to recognize and act upon potential errors [25]. This redesign will not only better prepare new graduates, but will help bridge the gap between education and current practice environment demands.

 Initial exposure to patient safety can occur early in undergraduate programs to create both safety problem and solution awareness and begin early professional formation that is inclusive of both safety and interprofessional teamwork frameworks. Students reported appreciating this education prior to entering clinical rotations as they felt better prepared and were more aware of safety as it tied to all professions. Findings from this study were similar to two previous examinations of the patient safety course, both from an overall perspective and looking specifically at pharmacy students. This further demonstrates the need for interprofessional patient safety education during the professional formation of health professionals in training [13, 14]. The call for the educational community to provide early learning to prepare for practice is critical. The authors and instructors of this interprofessional patient safety course believe that early introduction to these concepts and principles via academic education prior to practice allows students to make meaning in practice that can be internalized and acted upon when entering the workforce.

### 4.1. Limitations

 Interviews in this study were limited to second year students in a Bachelor of Science in Nursing program. Although they provided valuable insight into the possible benefits of providing interprofessional patient safety education early in the health professions curricula, it is plausible that those further into their programs would possess more refined viewpoints when it comes to their role as health care professionals, yet their lack of awareness of patient safety science would yield similar perceptions and observations as students in this study.

 Results of this study are also limited by the small number of students from only one nursing school. Overall, findings are limited to enriching our understanding of the value nursing students placed on the interprofessional study of patient safety. These findings are not intended to be representative of how the entire population of nursing students feels, but rather, they address an identified gap in our knowledge about the importance of interprofessional patient safety curricular content and further the discussion about how they may best be integrated into existing nursing education.

 This study was conducted as an elective course available to all health professions students. This may potentially bias these results as those students who have chosen to take this course may be more enthusiastic about the course content. It is important to note that although initial interest may dictate course selection, student engagement may diminish if the interest does not persist. The positive responses expressed at completion indicated that the content kept students engaged throughout.

### 4.2. Future Research/Implications for Education

 Findings from this study will inform educators about the meaning of interprofessional patient safety education experience to professional nursing students development. Students who have an awareness of patient safety principles may be able to act more readily in their clinical practice. This type of values-oriented patient safety education framework is responsive to nursing students and to workforce demands as well as it may enrich opportunities for nursing student education. Expanding this study to look at the value of this course to other disciplines taking the course and comparing them for similarities and differences would also be an area for future research.

## 5. Conclusion

 Interprofessional education is valuable to students as they learn and transfer into full-fledged practitioners. Gaining an understanding and an appreciation for the roles and responsibilities of others on the health care team is vital to achieving the goals of the IOM “Future of Nursing” report. Nursing educators in the United States need to structure nursing curricula to explicitly include opportunities for interprofessional interaction. One way to do this is through patient safety education that provides students with an understanding of principles that allow them to take ownership of their responsibilities within the health care team and act accordingly for the benefit of patients. Committed partnerships across professions are necessary for successful integration of interprofessional education. Through these endeavors, nurses can achieve their full practice potential.
